# Soil texture, infective juvenile concentration, and soil organic matter influence the efficacy of *Steinernema feltiae* isolate Lican Ray

**DOI:** 10.21307/jofnem-2020-007

**Published:** 2020-03-18

**Authors:** Gabriela Lankin, Giselle Vidal-Retes, Geraldine Allende, Carlos Castaneda-Alvarez, Ernesto San-Blas, Erwin Aballay

**Affiliations:** 1 Departamento de Sanidad Vegetal, Facultad de Ciencias Agronómicas, Universidad de Chile. P.O. Box 1004, Santiago, Chile; 2 Centro de Estudios Botánicos y Agroforestales, Instituto Venezolano de Investigaciones Científicas, Maracaibo, Venezuela

**Keywords:** Entomopathogenic nematodes, Clay soil, Loam soil, Organic matter, Sandy loam soil.

## Abstract

The influence of infective juveniles (IJs) concentration, soil texture, IJ-host distance and organic matter (OM) content, at different decomposition degree, on the activity of the nematode *Steinernema feltiae* isolate Lican Ray (LR) was examined using *Galleria mellonella* larvae. Bioassays were conducted in tubes of varied length, filled with soil of different textures, placed either vertically or horizontally. In the concentration assay, highest IJ concentrations caused maximum larval mortality in all soil types (440, 2,200 and 4,400 IJs in clay, loam and sandy loam). In the second assay, soil texture (loam, clay or sandy loam) interacted significantly with IJ-host distance (10, 20, 30, 40 cm, horizontally; 30, 50, 70 cm, vertically), and distances of 30 cm or more affected IJ effectiveness on the control of *G. mellonella.* The effect was stronger in clay and sandy loam than in loam soils, where IJ moved up to 40 cm horizontally and 70 cm vertically. In the third assay, OM content (0, 2, 4, 6 and 8%) and its decomposition degree (initial, medium and advanced) did not interact to influence IJ movement in all treatments that contained any percentage of OM (2–8%). Only in the soil with no OM, IJ did not cause death of larvae at all. These results show the potential of *S. feltiae* LR to be used in different soil textures, as long as the content of soil OM allows its dispersal and host infection, in order to optimize the pest-control activity of the nematode.

*Steinernema feltiae* (Nematoda: Rhabditidae) is a soil dwelling entomopathogenic nematode (EPN) that can exploit a wide range of insect hosts. The third-stage infective juvenile (IJ) locate insect hosts, enter their natural openings (mouth, anus or spiracles), and release symbiotic bacteria of the species *Xenorhabdus bovienii*, carried in their guts, into the host hemolymph causing septicemia and the death of the insect within 24–48 hr. The nematodes feed upon the proliferating bacteria and degraded host body tissues, producing two or more new generations within the insect cadaver before emerging into the soil as IJs in search of a new host. The potential of nematode of this genus for the control of several pest species has been demonstrated in Lepidoptera (Radhakrishnan et al., 2017), Coleoptera (Gulcu et al., 2019), Diptera (Chergui et al., 2019), among others. However, biotic factors such as antagonist organisms, abiotic factors, such as soil texture, moisture, temperature and content of organic matter (OM), and others, such as IJ concentration, application method and distance from the host, also affect the effectivity of the IJ (Choo and Kaya, 1991; Shapiro et al., 2000; Sharma et al., 2011; Griffin, 2015).

Regarding IJ concentration, many studies have focused on this factor for the control of different pest species (ranging from 0.1−1 × 10^6^ IJ m^−2^); however, adequate IJ concentration for different EPN species in various soil types is poorly understood. For example, to achieve a similar mortality level of *Phthorimaea operculella* (Lepidoptera: Gelechiidae) larvae, it was necessary to apply 500 IJ in sandy soil and 2,000 IJ in loam soil of either *Steinernema carpocapsae* or *Heterorhabditis bacteriophora* (Hassani-Kakhki et al., 2013). This is important to consider in commercial EPN applications, as an excessive or deficient IJ number applied may increase the costs unnecessarily or result in an inefficient control of the target pest.

In relation to soil texture, this factor affects dispersal (Kapranas et al., 2017), infectivity (Choo and Kaya, 1991; Toledo et al., 2009) and persistence (Shapiro et al., 2000; Koppenhöfer and Fuzy, 2006) of EPNs, which generally move and disperse better in light-textured soils (Hassani-Kakhki et al., 2013). For instance, *H. bacteriophora*, *S. carpocapsae* and *Steinernema glaseri* dispersed significantly more in sandy loam than in loam or silty clay loam (Portillo-Aguilar et al., 1999). Furthermore, this may vary with EPNs species and isolate, as in a study by Campos-Herrera and Gutiérrez (2014), where infection dynamics varied among 14 different populations of *S. feltiae* in the same soil texture. Besides, Campos-Herrera and Gutiérrez (2009) observed that heavy soils negatively affected the virulence of the Rioja isolate of *S. feltiae* on larvae of *Spodoptera littoralis* (Lepidoptera: Noctuidae), as an increase content of clay from 5 to 14%, caused an increase in the LC_90_ (to kill 90% of larvae in two days) from 220 to 4,178 IJs/cm^2^. Therefore, knowledge about soil requirements of an EPN species/isolate is critical to optimizing their performance as biological control agents in the field.

In addition, OM improves soil structure, lowering bulk density, increasing the available space through the distribution of aggregates and pore size, thus OM content and soil compaction may influence EPNs infection patterns (Kapranas et al., 2017), and persistence in the soil (Shapiro and Lewis, 1999). While several studies have shown the benefits of soil OM on soil inhabitants in general (Hoitink and Boehm, 1999; Davey et al., 2019), few have focused on EPN performance (Kapranas et al., 2017). For instance, Herren et al. (2018) observed higher survival and virulence of *S. feltiae* on larvae of *Galleria mellonella* (Lepidoptera: Pyralidae) in soils with added mature compost. Some authors propose that the compost could be used as a carrier for EPN application in the field (Herren et al., 2018; Georgis et al., 2006) as it could protect them against UV radiation, extreme temperatures and facilitate contact between EPNs and their insect hosts (Georgis et al., 2006). Therefore, texture, OM and IJ concentration are factors to consider for optimizing EPN activity, and they should be evaluated for different species.

The native Chilean EPN *S. feltiae* isolate Lican Ray (LR) was successfully evaluated, under laboratory and semifield conditions, for the control of foliage pests like the diamond back moth, *Plutella xylostella* (Lepidoptera: Plutellidae) (Reyes, 2018; Nuñez, 2017) and the bagrada bug, *Bagrada hilaris* (Hemiptera: Pentatomidae) (unpublished data). Also on soil pests, such as the potato cutworm *Agrotis deprivata* (Syn. *A. bilitura*) (Lepidoptera: Noctuidae) (Burgos, 2017; Quezada, 2018) and larvae of *Naupactus xanthographus* (Coleoptera: Curculionidae) (unpublished data). Considering the great potential of *S. feltiae* LR for the control of these and other pests, the objectives of this work were: to evaluate the infectivity of this isolate on *G. mellonella* larvae at three IJ concentrations in clay, loam and sandy loam soils; to assess the IJ capacity to disperse (horizontally and vertically), reach and kill *G. mellonella* larvae in these soils; and to determine the effect of different OM concentrations and different degrees of decomposition on the dispersion of IJs in loam soil.

## Materials and methods

IJs of *S. feltiae* LR were cultured at 25 ± 0.1°C using last larval instars of *G. mellonella* (Kaya and Stock, 1997), and IJs were stored at 8°C in tissue culture flasks with distilled water for less than five days before their use.

Three different soils were used in the experiments. Sandy loam and clay soils were obtained from Pichidegua town, located in the Libertador Bernardo O´Higgins Region, Chile. Loam soil was obtained from Universidad de Chile’s experimental field located at La Pintana, in the Metropolitan Region, Santiago, Chile.

The clay soil was sieved, and all soils were sterilized in metallic cans using the water bath method at 90°C for 2 hr, and then physical and chemical parameters of the soil samples were determined in the Laboratory of Soil from Universidad de Chile (Table 1). The samples were moistened up to field capacity and stored in closed plastic containers at 25 ± 2°C for up to 48 hr before use.

**Table 1.** Physical properties of the soils used.

	Volumetric water content (%)			Texture (%)		
Textural classes	FC	PWP	AWC	S	C	Si
Clay	39.0	19.0	20.0	19.0	52.0	19.0
Sandy loam	18.0	8.9	9.1	69.2	8.6	22.2
Loam	22.0	14.0	8.0	38.8	20.8	40.4

Notes: FC, field capacity; PWP, permanent wilting point; AWC, available water content; Texture according to the content of Sand (S), Clay (C) and Silt (Si).

OM in the form of Vermicompost was obtained from a vermiculture farm, which used rests of vegetables from the kitchen of a cafeteria. Three types of vermicompost were used according to their age and degree of decomposition: initial (less than one month), medium (three months) and advanced (six months). All the samples were sterilized, as described previously, and prior to use, the vermicompost was sieved (3 mm opening for initial; 2 mm for others), chemical parameters determined as above (Table 2), and stored at −80°C to stop the decomposition process. On the day of the assay, it was moistened up to field capacity.

**Table 2.** Chemical analysis of organic matter (OM) used, at different degrees of decomposition.

	Content (%)			Content (ppm)	
OM decomposition degree	OM	Organic carbon	Total nitrogen	Nitrate	Ammonium
Initial	85	49.7	2.66	147	142
Medium	18	10.5	1.26	495	28
Advanced	18	10.5	0.98	335	18

**Figure 1:**
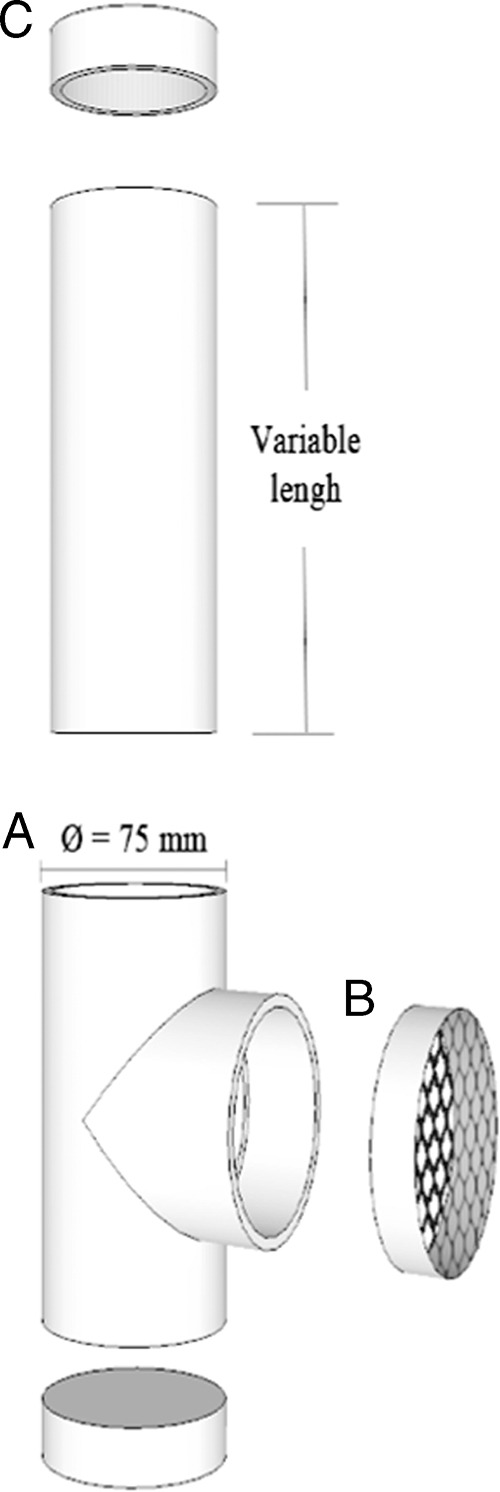
Through the lateral opening of this t-shaped PVC matrix (A), a modified Petri dish containing soil and larvae is introduced. A removable lid with a metallic mesh for gas exchange covers the lateral opening (B). IJs are pipetted through the upper opening (C).

Bioassays were conducted using a 75 mm diameter and 25 cm long t-shaped PVC tube, denominated matrix (Fig. 1A), filled with soil. Through the lateral opening (Fig. 1B), a modified 60 mm diameter plastic petri dish was introduced. This dish had both sides replaced by 1 mm stainless steel mesh, and contained soil corresponding to the treatment, and five last larval instars of *G. mellonella*. Removable lids closed the end matrix openings, and the lateral lid was modified with a metallic mesh for gas exchange (Fig. 1B). According to the treatments, matrices were filled with different soil type, previously sterilized and moistened.

### Optimal concentration of *S. feltiae* LR in three soil types

The matrices were placed in vertical position and through the upper opening IJs were pipetted in one of the following suspension 0, 440, 2,200 or 4,400 IJs/5 mL water (Sharma et al., 2011). The distance between the petri dishes and the inoculation point was 20 cm. Each type of soil in combination with each IJ concentration defined the treatments (12 treatments), with five repetitions each. The matrices were maintained vertically in darkness at 25 ± 2°C. Three, six and nine days after inoculation (DAI), the petri dishes were removed, and larval mortality assessed.

### Horizontal and vertical dispersal capacity of *S. feltiae* LR in three soil types

For this assay, another tube (Fig. 1C) of variable length according to the treatment, was adapted in the upper opening of the matrix, resulting in tubes with soil columns (distance between inoculation point and petri dishes) of total length of 10, 20, 30 and 40 cm for evaluating horizontal movement and 30, 50 and 70 cm for evaluating vertical movement of EPNs. The matrices were placed either in vertical or horizontal position, and then inoculated, with 4,400 IJs in 5 mL of water (the best concentration in the previous experiment) through the upper opening, closed and incubated as previously described. Combining the factors (soil type × distance) resulted in 12 (3 × 4) and 9 (3 × 3) treatments in horizontal and vertical assays, respectively, with five repetitions each. Three, six and nine days after inoculation (DAI), the petri dishes were removed, and larval mortality assessed.

### Effect of OM at different decomposition degree on the dispersal of *S. feltiae* LR

Two, 4 and 6% v/v OM at different decomposition degree was added to loam soil with a natural content of 2% OM (the soil with best results in Assay 2) obtaining final concentrations of 4, 6 and 8%. In addition, two control treatments were set, the first consisted on the same soil with no OM added (natural content 2%) and the second, the same soil but eliminating OM by calcination in a muffle furnace (LabTech^®^, Korea) at 360°C for 16 hr. Finally, each matrix (containing a soil column of 20 cm) was inoculated with 5 mL of a suspension of 4,400 IJs. The combination of the factors (percentage of OM × decomposition degree) and the control treatments resulted in 11 treatments, repeated five times each. Two and four days after inoculation (DAI), the petri dishes were removed, and larval mortality assessed.

In all the three assays the movement capacity of IJs was evaluated through the mortality level of the insect larvae, as IJs were capable of reaching and infecting the host (*G. mellonella*) at certain distances. In all cases dead larvae were washed with distilled water and placed in white traps (Kaya and Stock, 1997) at 25°C until IJ emergence occurred. In all assays, sentinel treatments, consisting on five *G. mellonella* larvae inoculated in petri dish with 500 IJ, were left in the chamber at the same temperature, to verify EPN viability and correct handling of the assay.

### Experimental design and statistic analysis

All assays were conducted under laboratory conditions and arranged in a randomized block design with factorial structure. Petri dishes with *G. mellonella* larvae were considered as the experimental unit. Percentage of mortality was angular transformed and analyzed through general linear model. Data in Assay 1 were corrected by the Schneider-Orelli’s formula (Püntener, 1981). When *F* values were significant, differences between means were evaluated using Tukey’s multiple range (*p* ≤ 0.05). Minitab^®^ V17 was used for data analysis. All assays were repeated twice.

## Results

### Optimal concentration of *S. feltiae* LR in three soil types

**Figure 2:**
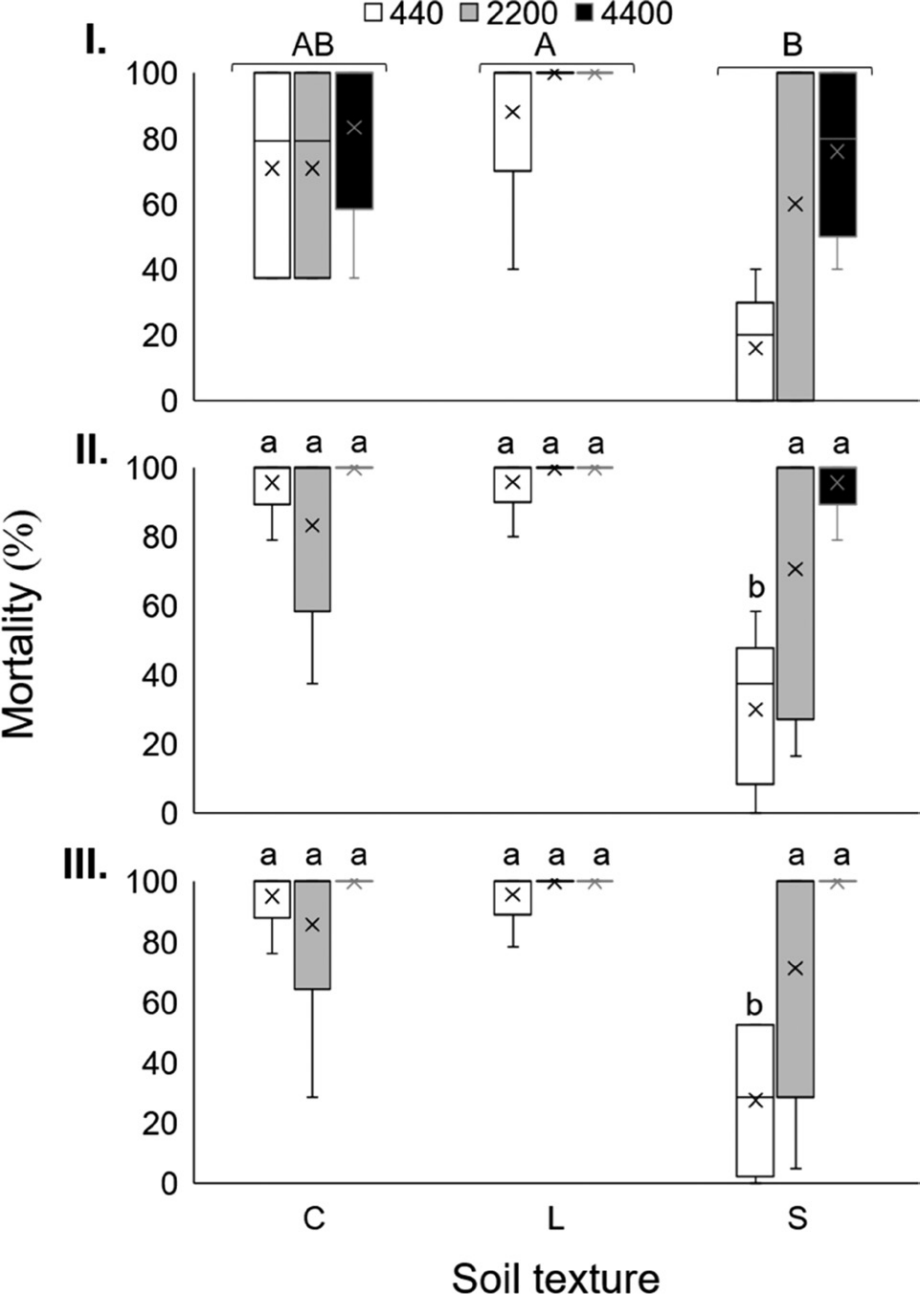
Boxplot showing percentage of last instar larvae *Galleria mellonella* mortality at (I) three, (II) six and (III) nine DAI, inoculated with 440, 2,200 or 4,400 IJs/5 mL of *Steinernema feltiae* LR in clay (C), loam (L) or sandy loam (S) soil, corrected according to the Schneider-Orelli’s formula). In (I) bars with different capital letters indicate statistical differences for soil texture (Tukey’s test; *p* ≤ 0.05). In (II) and (III) bars with different lower-case letter indicates statistical differences for the interaction between IJ concentration and soil texture (Tukey’s test; *p* ≤ 0.05). In Figs. 2–4, boxes represent the third quartile (75th percentile), median (50th percentile), first quartile (25th percentile), and mean percentage (x) of five replicates, with upper whiskers reaching Q3 +1.5×interquartile range (IQR) and lower whiskers Q1−1.5 × IQR.

At three DAI the analysis showed no interaction between IJ concentration and soil types (*F*_(4,36)_ = 1.13; *p* = 0.357) therefore, factors were analyzed independently. Considering the factor soil texture, and independently from the IJ concentration, the highest *G. mellonella* mortality occurred in loam soil (96%), followed by clay soil (75%), and sandy soil (50.7%), and significant differences were observed only between loam and sandy soils (*F*_(2,36)_ = 9.59; *p* ≤ 0.0001; Fig. 2I, capital letters). However, considering the factor IJ concentration and regardless of soil texture, no significant differences were observed between treatments at this time (*F*_(2,36)_ = 3.23; *p* = 0.051) (therefore, no lower-case letters on bars, Fig. 2I).

At six DAI the analysis showed significant interactions between IJ concentration and soil type (*F*_(4,36)_ = 4.59; *p* = 0.004), with significantly lowest *G. mellonella* mortality in sandy soils at the concentration of 440 IJs (30%) compared to all other treatments, where mortality varied between 70.8 and 100% (Fig. 2II). A similar situation occurred at nine DAI (*F*_(4,36)_ = 4.91; *p* = 0.003), where mortality varied between 71.4 and 100%, with significantly lowest mortality in sandy soils and 440 IJ (Fig. 2III).

At the end of the experimental period (nine DAI), 4,400 was the only IJ concentration that achieved 100% of larval mortality in all soil types, therefore, this concentration was used in further assays.

### Horizontal and vertical dispersal capacity of *S. feltiae* LR in three soil types

In all evaluation times the interaction between the factors soil texture and distance was significant, affecting IJ capacity to reach the host and infect it. The general trend was that an increase in the distance resulted in lower mortality; however, particular variations were observed according to soil type and matrix position.

#### Horizontal movement

**Figure 3:**
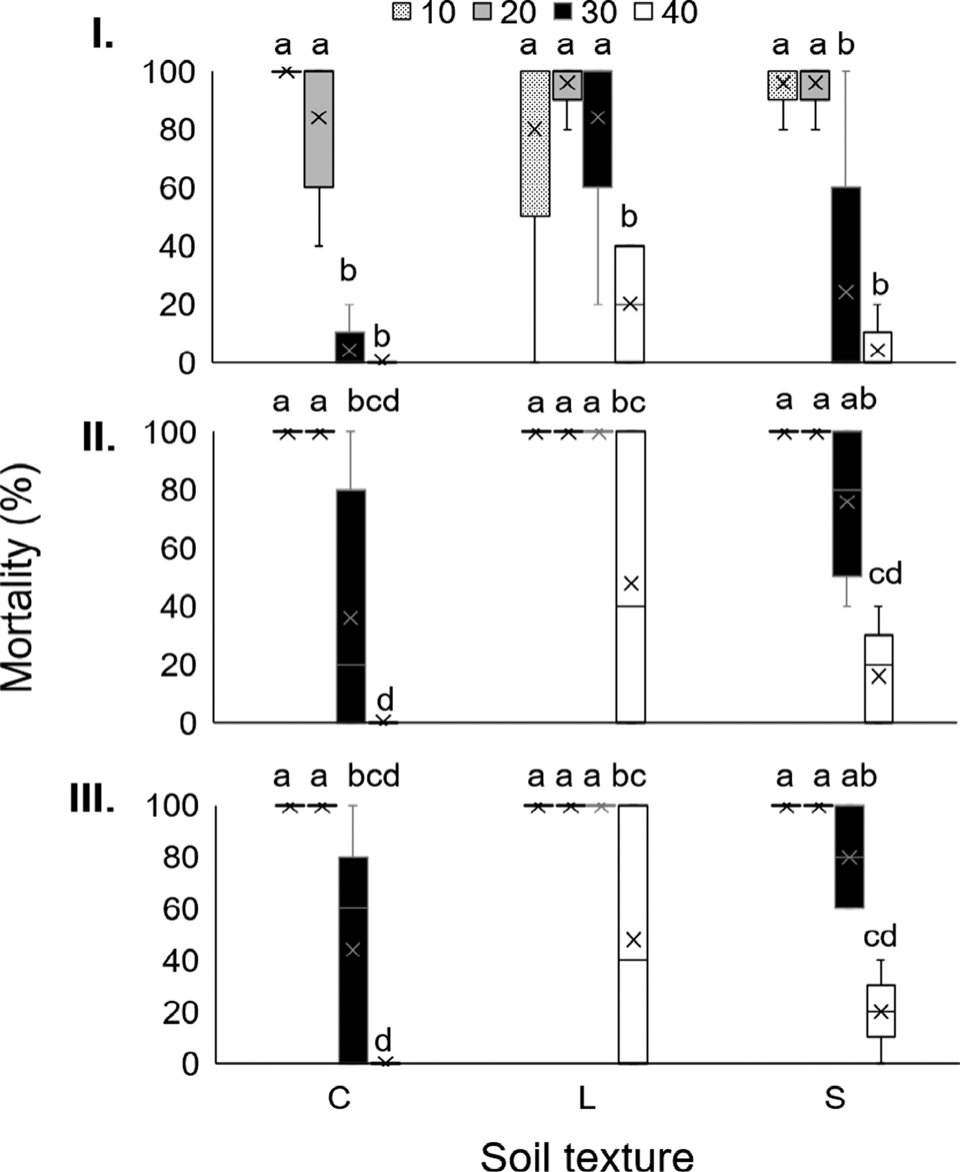
Boxplot showing percentage of last instar larvae *Galleria mellonella* mortality at a distance of 10, 20, 30 or 40 cm horizontally from the inoculation point of IJ of *Steinernema feltiae* LR in clay (C), loam (L) or sandy loam (S) soil at (I) three, (II) six and (III) nine DAI. Bars with different letter in each evaluation time, indicate statistical differences, according to Tukey’s test (*p* ≤ 0.05).

At three DAI (*F*_(6, 48)_ = 4.21; *p* = 0.002), when larvae were 10 and 20 cm from the IJ inoculation point, in all soil textures, and 30 cm in loamy soil, mortality reached significantly highest values (80–100%). The lowest mortality levels were observed at 30 and 40 cm in most soils (approximately 0–20%) (Fig. 3).

At six DAI (*F*_(6, 48)_ = 3.07; *p* = 0.013) and nine DAI (*F*_(6, 48)_ = 2.84; *p* = 0.019), significantly highest mortality was recorded at 10 and 20 in all soil textures, at 30 cm in loam soil (100%) and 30 cm in sandy soil (75%), followed by clay and sandy at 30 cm, and loam soil at 40 (35–75%). The lowest larval mortality occurred in clay and sandy soil at 40 cm (Fig. 3).

#### Vertical movement

**Figure 4:**
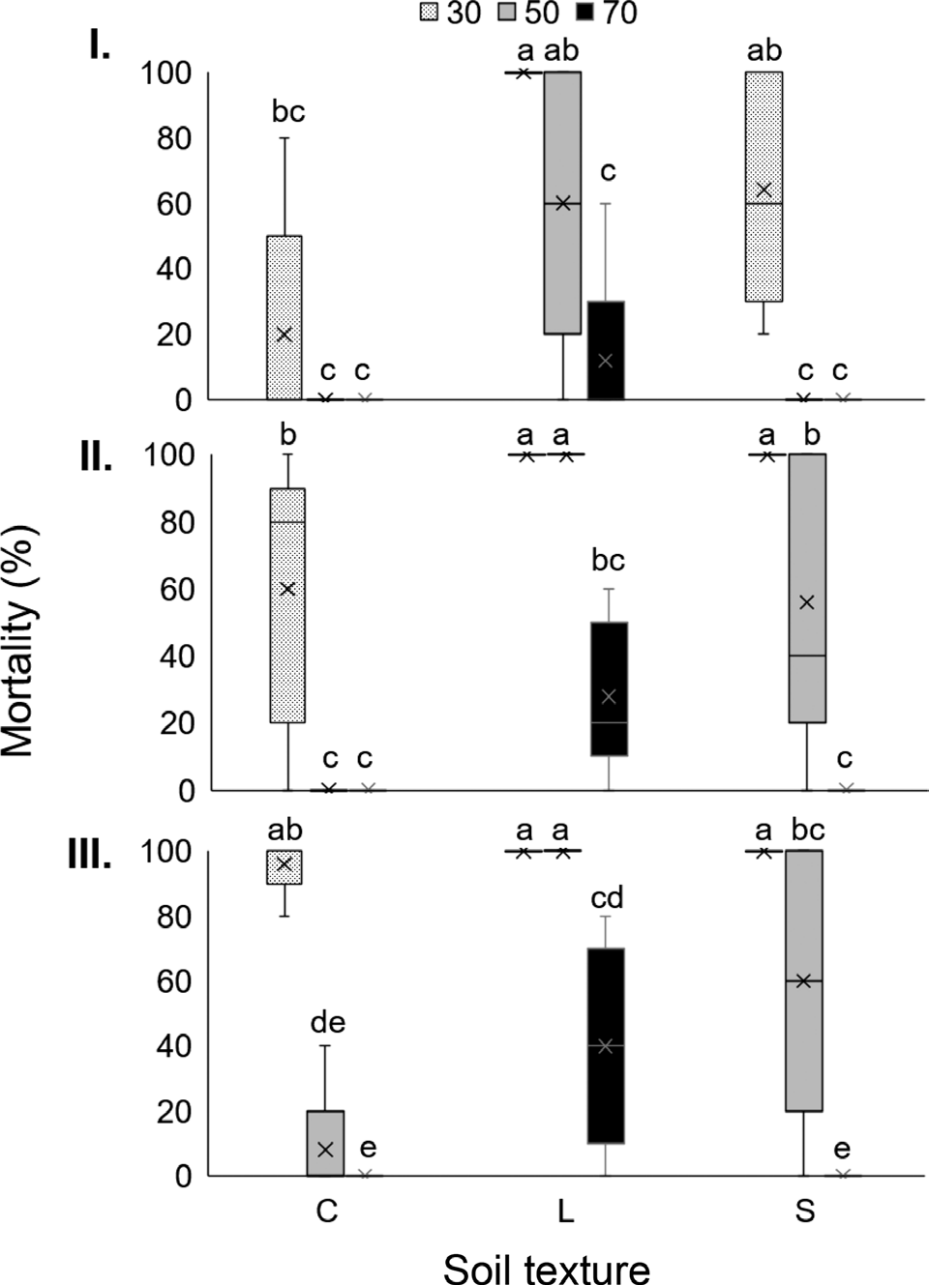
Boxplot showing percentage of last instar larvae *Galleria mellonella* mortality at a distance of 30, 50 or 70 cm vertically from the inoculation point of IJ of *Steinernema feltiae* LR in clay (C), loam (L) or sandy loam (S) soil at (I) three, six (II) and (III) nine DAI. Bars with different letter in each evaluation time, indicate statistical differences, according to Tukey’s test (*p* ≤ 0.05).

At three DAI (*F*_(4, 36)_ = 4.31; *p* = 0.006), highest mortality was recorded in loam soil at 30 cm (100%) and 50 cm (60%), and in sandy loam soil at 30 cm (64%). The lowest mortality level was observed at 50 and 70 cm in most soils (approximately 0–12%) (Fig. 4).

At six DAI (*F*_(4, 36)_ = 5.54; *p* = 0.001) and nine DAI (*F*_(4, 36)_ = 7.78; *p* < 0.001), significantly highest mortality was recorded in loam soil at 30 and 50 cm and in sandy soil at 30 cm (100%), followed by clay at 30 cm, sandy at 50 cm and loam soil at 70 cm. In addition, at six DAI there was no larval mortality at 50 and 70 cm in clay, and at 70 cm in sandy soils. Finally, in clay soils, at nine DAI, mortality tended to increase at 30 and 50 cm compared to six DAI (Fig. 4).

### Effect of OM at different decomposition degree on the dispersal of *S. feltiae* LR

**Figure 5:**
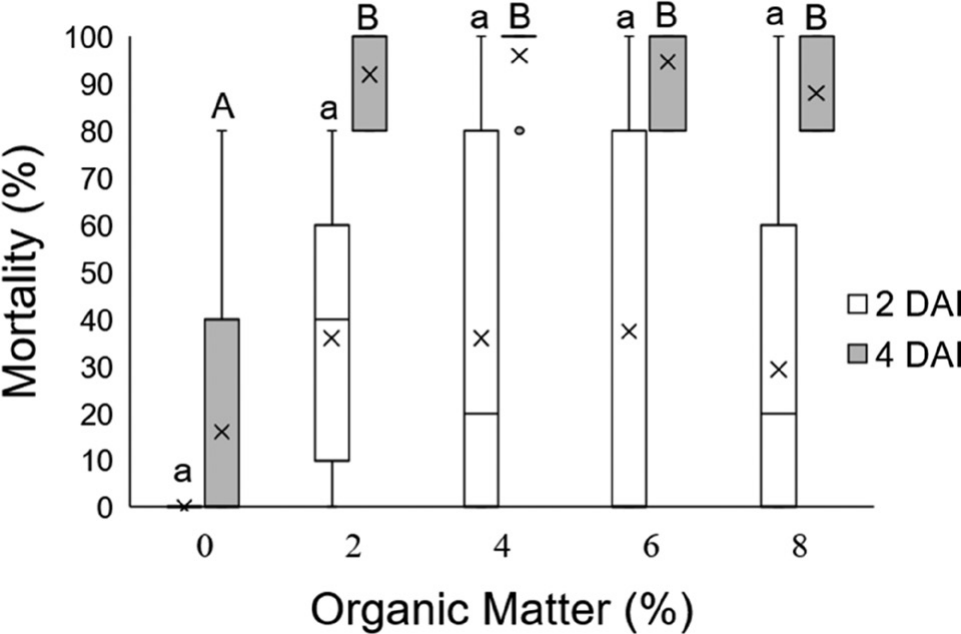
Boxplots showing percentage of last instar larvae *Galleria mellonella* mortality caused by *Steinernema feltiae* LR in loam soil at two and four DAI with 0, 2, 4, 6 or 8% OM. Bars with different letters indicate statistical differences, according to Tukey’s test (*p* ≤ 0.05) (lower case for two DAI and capital letters for four DAI). In figs 5 and 6, boxes represent the third quartile (75th percentile), median (50th percentile), first quartile (25th percentile), and mean percentage (x) of 15 replicates, with upper whiskers reaching Q3 + 1.5 × interquartile range (IQR) and lower whiskers Q1−1.5 × IQR. Outliers beyond the boxes limits are plotted as individual points.

**Figure 6:**
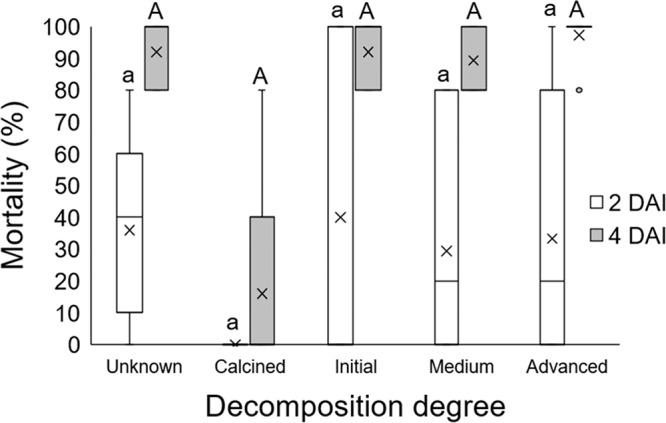
Boxplots showing percentage of last instar larvae *Galleria mellonella* mortality caused by *Steinernema feltiae* LR in loam soil at two and four DAI with OM at different decomposition degrees (initial, medium and advanced) control soil (unknown) and soil with no OM (calcined). Bars with different letters on bars indicate statistical differences, according to Tukey’s test (*p* ≤ 0.05) (lower case for two DAI and capital letters for four DAI).

At two DAI (*F*_(4, 40)_ = 0.14; *p* = 0.965) and four DAI (*F*_(4, 40)_ = 0.53; *p* = 0.718), the interaction between the factors OM content and OM decomposition degree was no significant, therefore, these factors were analyzed independently in relation to their effect on the IJ capacity to reach the host and infect it. On one hand, considering the factor OM content, at two DAI (Fig. 5, lower-case letters) the only treatment where no mortality was observed was the soil with no OM. In general, in soils with OM (2–8%) larval mortality increased with OM content, however, these differences were not significant among all treatments. At four DAI (Fig. 5, capital letters) larval mortality was significantly higher in all treatments with OM (88–96%) compared to the calcined soil (16%). On the other hand, considering the factor OM decomposition degree, the independent analysis resulted in the comparison of five treatments: original soil with unknown OM decomposition degree; calcined soil without OM; soils with initial; medium; and advanced degree of OM decomposition. At two DAI, except for the soil with no OM, all treatments had a similar larval mortality level of approximately 34%, however these differences were not significant. Similar results were obtained at four DAI, with mortality near 93% for all treatments with OM, which was higher than the treatment with no OM, but this difference was no significant (Fig. 6).

## Discussion

The *S. feltiae* isolate LR evaluated in this work displayed the expected dispersal behavior in the soil, according to *S. feltiae* foraging strategy (Alekseev et al., 2006; Campos-Herrera and Gutiérrez, 2014). The geographic diversity of Chile (from 18 °S latitude to 56 °S latitude) results in a myriad of soils under different management strategies, and biotic and abiotic characteristics. In this work, we verified the potential of this native isolate to be used under different conditions for the control of soils pests.

Although IJ concentration for the control of different pests have been widely studied (Gazit et al., 2000; Batista et al., 2014; Guo et al., 2017), the influence of soil texture at different IJ concentrations on pest control is poorly understood. For instance, Koppenhöfer and Fuzy (2006) found that the highest concentration of *Steinernema scarabaei* caused the highest mortality levels on *Anomala orientalis* (Coleoptera: Scarabeidae) in all substrates studied (loamy sand, sandy loam, loam, silt loam, acidic sand and potting mix). In contrast, in a study by Campos-Herrera and Gutiérrez (2009), the infectivity of *S. feltiae* isolate the Rioja against *S. littoralis* was affected by both soil type and IJ dosage, with higher mortality achieved with higher IJ doses in heavier soils. However, in these soils, the number of days to larval death was highest. Similarly, we observed that IJ dosage and soil texture affect IJ movement and its capacity to reach and kill the insect host. In our study, initially, the highest levels of mortality were achieved in loam soil, regardless of the IJ concentration, achieving the highest levels of mortality at six DAI. However, as time passed, both texture and IJ concentration influenced larval mortality, particularly in light-textured soil (sandy loam), where there was a clear tendency to higher mortality as the concentration increased.

These results suggest that in field conditions, higher IJ concentrations of *S. feltiae* should be utilized in applications for light soils. In heavy soils (clay), even though no significant differences were observed, the highest mortality was achieved with the highest IJ concentration, but it is likely that the percentage of clay in the soil will define the feasibility for EPN to disperse. According to Wallace (1968), the nematodes are confined to the existing soil geometry, and pore size correlates with movement; nematodes cannot pass through spaces much narrower than their body diameter. Therefore, the isolate selected should consider its body size and soil clay content.

Several studies have shown that soil texture affects the capacity of EPN to disperse and infect the host, such as *Steinernema riobrave* and *H. bacteriophora* on *Diaprepes abbreviatus* (Coleoptera: Curculionidae) (Shapiro et al., 2000), *H. bacteriophora*, *H. megidis* and *S. feltiae* on *Diabrotica virgifera virgifera* (Coleoptera: Chrysomelidae) (Toepfer et al., 2010), and *S. carpocapsae* and *H. bacteriophora* on potato tuber moth, *P. operculella* (Hassani-Kakhki et al., 2013). In some cases, light textures were the most beneficial for various EPNs species (Hassani-Kakhki et al., 2013).

However, in our study, the native EPN, *S. feltiae* LR, was capable to disperse more in intermediate (loam), both vertically and horizontally. This isolate moved through the soil and reached the host at a distance of 30 cm horizontally in all soil types evaluated, except in loam, where they were able to move up to 40 cm. Furthermore, they achieved vertical distances of 70, 50 and 30 cm in loam, sandy loam and clay soil, respectively. Other studies found similar results with vertical movement of *S. feltiae* on the host *G. mellonella* (Neumann and Shields, 2006), also of *S. carpocapsae* on *Anastrepha obliqua* (Diptera: Tephritidae) (Toledo et al., 2009), and vertical and horizontal of *Heterorhabdits amazonensis* and *Steinernema arenarium* on *Spodoptera frugiperda* (Lepidopera: Noctuidae) (Andaló et al., 2012). In addition, in our study, mortality of *G. mellonella* decreased with the extreme soil textures evaluated. Horizontally at 30 cm, while in loam soil mortality reached 100% at the end of the experimental period, in sandy and clay soil it reached 80 and 45%, respectively. Similarly, at 40 cm, while in loam soil mortality reached 50%, in sandy and clay soil it reached only 20 and 0%, respectively.

However, the results have been diverse regarding which soil texture benefits EPNs activity, as the soil pore size can also affect IJ activity in the soil, because energy reserves are depleted with more movement. For example, smaller pores result in higher opposition to IJ movement and/or less oxygen available and consequently, IJs may lose efficacy finding, reaching and infecting their hosts (Wallace, 1968). Native isolates, even from the same species, may show better performance in specific soil textures. For example, in a study by Toepfer et al. (2010), the efficacy of three EPNs species, including *S. feltiae*, on *D. v. virgifera* larvae was higher in maize fields with heavy clay or silty clay soils than in sandy soils. In addition, *S. carpocapsae* and *S. riobrave* moved more easily in sandy loam and marine sand soils (Alekseev et al., 2006), and *S. riobrave* and *H. bacteriophora* dispersed more easily in sandy soils (Shapiro et al., 2000). Our study showed greater infective capacity efficacy of IJ in soils with medium pore size, however, other species, such as *S. riobrave* and *H. bacteriophora*, showed greater infection capacity on *D. abbreviatus* and longer persistence in Marl soil (80% silt and 15% clay) compared to sandy soil (Shapiro et al., 2000). Likewise, *S. carpocapsae* showed the highest percentage of infection on *A. obliqua* larvae in sand-clay mixture (60–82% depending on depth), compared to 45–64% in sand, and 23–30% in loamy-sand soil (Toledo et al., 2009).

Therefore, light soils (containing more sand and less clay) do not necessarily guarantee IJs efficacy, because there may be a strong relationship between soil pores and IJ size, which can benefit IJ dispersal. The dispersal rate in our study was higher in loam soil compared to the other textures, where IJs of *S. feltiae* LR reached *G. mellonella* (a non-mobile host), located 30 and 50 cm horizontally and vertically, respectively, in only three days. Similar studies were conducted by Bal and Grewal (2015) where *S. carpocapsae* and *H. bacteriophora*, moved horizontally approximately 15 and 10 cm in the same period, reaching a non-mobile host and 24 and 15 cm with a mobile host, respectively, in silt-loamy soil. However, further studies by Bal et al. (2017) showed that the same species moved an estimate of 150 cm in 3 days, under field conditions. Therefore, it is crucial to define the adequate EPN isolate for each soil type, which optimizes dispersal and time to reach the host, factors that should be considered together with IJ concentration in further field studies.

Although soil structure may be improved with the addition of OM, in this work, structure was not considered, because it was altered in the process of mixing the soil with OM and filling the tubes. Only the content of OM and the decomposition degree were considered. While EPN movement in organic substrate has received little attention, Griffin (2015), Ansari and Butt (2011) and Kapranas et al. (2017) point out that EPN behavior is influenced by the species and soil quality. For instance, both compaction and increasing peat content in sand, decreased IJ success in *S. carpocapsae*, *Heterorhabditis downesi* and *S. feltiae* (Kapranas et al., 2017). Also, according to Williams et al. (2013), it is likely that organic soils facilitate the active or passive movement of nematodes through the medium or promote their post-application survival. For example, Kruitbos et al. (2010) observed that while *S. carpocapsae* moved significantly less in sand than in peat, *H. megidis* dispersed well in both media, but only showed taxis toward hosts in sand. In addition, MacMillan et al. (2009) observed a more active dispersal of two *Phasmarhabditis hermaphrodita* strains in organic media (bark chips and leaf litter, and to a lesser extent peat) than in mineral soils. Similarly, our study evidenced, first, that the OM content influences IJ movement of *S. feltiae* LR, especially those treatments with OM content different from zero. Even though no significant differences in *G. mellonella* mortality were observed in the treatments that contained OM (2–8%), our results suggest that 4% would be the optimal content for this EPN and values above or below this, affect its capacity to reach and kill the host. Second, we observed that the OM decomposition degree (particle size), does not influence the capacity of *S. feltiae* LR to reach and kill the host. Regarding survival of the EPN, Herren et al. (2018) observed higher survival of *S. feltiae* in mature compost that in soil with no compost added; however, this was also affected by the application mode of the EPN. In their work, the survival ranged from only 3.2%, when EPNs were applied in aqueous suspension to soils with no compost added, to 62% when they were applied in clay-coated *G. mellonella* cadavers on soil with mature compost added.

Even though *S. feltiae* is a well-studied species, and several companies around the world have used it to formulate products commercially available, EPN intra-species variability concerning phenotypical and biological behavior has been demonstrated (Gaugler et al., 1989; Campos-Herrera and Gutiérrez, 2014). Therefore, when developing a product based on native EPN, it is important to study specific characteristics of these ecotypes to optimize its use under local conditions. Further studies under field conditions, using a wider range of soil OM content and including those textures that affected negatively the movement of *S. feltiae* LR (extreme textures), which could be improved adding OM, should be also considered.
